# Role of truncated oxidized phospholipids in acute endothelial barrier dysfunction caused by particulate matter

**DOI:** 10.1371/journal.pone.0206251

**Published:** 2018-11-12

**Authors:** Pratap Karki, Angelo Meliton, Alok Shah, Yufeng Tian, Tomomi Ohmura, Nicolene Sarich, Anna A. Birukova, Konstantin G. Birukov

**Affiliations:** 1 Department of Medicine, University of Maryland, Baltimore, Maryland, United States of America; 2 Section of Pulmonary and Critical Care Medicine, Department of Medicine, University of Chicago, Chicago, Illinois, United States of America; 3 Department of Anesthesiology, University of Maryland, Baltimore, Maryland, United States of America; Emory University School of Medicine, UNITED STATES

## Abstract

Particulate matter (PM) air pollution is a global environmental health problem contributing to more severe lung inflammation and injury. However, the molecular and cellular mechanisms of PM-induced exacerbation of lung barrier dysfunction and injury are not well understood. In the current study, we tested a hypothesis that PM exacerbates vascular barrier dysfunction via ROS-induced generation of truncated oxidized phospholipids (Tr-OxPLs). Treatment of human pulmonary endothelial cells with PM caused endothelial cell barrier disruption in a dose-dependent fashion. Biochemical analysis showed destabilization of cell junctions by PM via tyrosine phosphorylation and internalization of VE-cadherin. These events were accompanied by PM-induced generation of Tr-OxPLs, detected by mass spectrometry analysis. Furthermore, purified Tr-OxPLs: POVPC, PGPC and lyso-PC alone, caused a rapid increase in endothelial permeability and augmented pulmonary endothelial barrier dysfunction induced by submaximal doses of PM. In support of a role of TR-OxPLs-dependent mechanism in mediation of PM effects, ectopic expression of intracellular type 2 platelet-activating factor acetylhydrolase (PAFAH2), which specifically hydrolyzes Tr-OxPLs, significantly attenuated PM-induced endothelial hyperpermeability. In summary, this study uncovered a novel mechanism of PM-induced sustained dysfunction of pulmonary endothelial cell barrier which is driven by PM-induced generation of truncated products of phospholipid oxidation causing destabilization of cell junctions.

## Introduction

Particulate matter (PM) is the most common air pollutant with a serious global health threat contributing to millions of premature deaths annually worldwide. The role of PM air pollution in the development or exacerbation of various heart and lung diseases is becoming increasingly recognized [1–3]. Among these diseases, two third include cardiovascular diseases such as ischemic heart disease, congestive heart failure etc. and one third are respiratory illnesses such as chronic obstructive pulmonary disease, acute lower respiratory tract infections, pneumonia, asthma and lung cancer [3–8]. PM is also suggested to have indirect detrimental effects on cerebrovascular as well as reproductive and developmental health. In turn, PM directly impacts the development and function of the lung [9, 10]. One of the well described mechanisms of PM pathological effects on lung is the induction of lung inflammation accompanied by endothelial cell (EC) dysfunction [11–13]. PM is known to cause thrombosis in an inflammation-dependent manner via the generation of IL-6 [14, 15]. PM exposure also generates reactive oxygen species (ROS) that plays a critical role in PM-induced cardiopulmonary disorders [15, 16]. In the longer term, PM exposure induces epigenetic modifications especially DNA methylation and controls the expression of various inflammatory and oxidant stress-related genes [17–20].

The endothelial barrier formed by the cell-cell junction complexes controls the passage of fluids, solutes and circulating cells across the vascular wall. Compromised EC barrier integrity leads to the excessive leakage and development of life-threatening conditions such as pulmonary edema and sepsis [21]. Adherens junctions play a major role in control of EC permeability; and transmembrane protein VE-cadherin is a central player in the formation of adherens junctions and regulation of endothelial barrier integrity. VE-cadherin is linked to actin cytoskeleton by its interactions with catenin family of proteins (α, β, γ and p120-catenins) [22]. The disassembly of VE-cadherin-catenin complex following tyrosine phosphorylation, internalization or cleavage of VE-cadherin causes the destabilization of AJ with an increase in endothelial permeability [23–26]. Exposure of endothelial cells to PM has been suggested to disrupt endothelial barrier function [27, 28], but the underlying mechanisms still remain to be fully elucidated. Oxidant stress, generation of reactive oxidant species (ROS) and subsequent activation of MAP kinases and Rho pathways have been suggested to mediate PM-induced endothelial barrier dysfunction [27, 29].

Phospholipids provide the structural basis for the formation of cell membranes. In turn, phospholipid oxidation generates bioactive lipid mediators that play an important role in cellular signaling [30, 31]. The increased accumulation of oxidized phospholipids from enzymatic or non-enzymatic cleavage of phospholipids have been reported in many pathological conditions including atherosclerosis, auto immune disease, lung injury and sepsis [30]. Among these, 1-palmitoyl-2-arachidonoyl-*sn*-glycero-3-phosphorylcholine (PAPC) represents the major plasma membrane phospholipid and its oxidation generates a heterogeneous mixture of oxygenated full-length products such as 1-palmitoyl-2-(5,6-epoxyisoprostane E2)-*sn*-glycero-3-phsphocholine (5,6-PEIPC), 1-palmitoyl-2-(5,6-epoxycyclopentenone)-*sn*-glycero-3-phsphocholine (5,6-PECPC); and truncated products of PAPC oxidation including 1-palmitoyl-2-(5-oxovaleroyl)-*sn*-glycero-phosphocholine (POVPC), 1-palmitoyl-2-glutaroyl-*sn*-glycero-phosphocholine (PGPC) and lysophosphatidyl choline (lyso-PC). Importantly, these truncated oxidized phospholipids (Tr-OxPLs) have been shown to induce endothelial barrier disruption [32].

In this study, we tested the hypothesis that PM challenge triggers the production of bioactive Tr-OxPLs by pulmonary EC, which cause AJ breakdown and endothelial barrier dysfunction. We also evaluated potential molecular approaches to reduce the levels of Tr-OxPLs and mitigate barrier disruptive effects of PM on pulmonary vascular endothelium.

## Materials and methods

### Cell culture

Human pulmonary artery endothelial cells and endothelial growth media were obtained from Lonza (Allendale, NJ). Cells were used at passages 5–8 and all cell stimulations were carried out in medium containing 2% fetal bovine serum unless otherwise specified. Texas Red-conjugated phalloidin and Alexa Fluor 488-labeled secondary antibodies were purchased form Molecular Probes (Eugene, OR). Antibodies to phospho-VE-cadherin (pTyr-658 and pTyr-731) were obtained from Invitrogen (Carlsbad, CA) and VE-cadherin antibody was from Santa Cruz Biotechnology (San Jose, CA). p120-Catenin antibody was from BD biosciences (San Diego, CA) and HRP-linked anti-mouse and anti-rabbit IgG were obtained from Cell Signaling (Beverly, MA). N-acetyl cysteine and amifostine were obtained from Sigma (St. Louis, MO). For PM, we used an urban PM 1649b collected from ambient air in Washington, DC and characterized by National Institute of Standards and Technology (certification date: 12/17/2015; expiration date: 07/31/2030). Purified POVPC, PGPC and lyso-PC were obtained from Avanti Polar Lipids (Alabaster, AL).

### ROS measurement

Human pulmonary endothelial cells were grown in 96-well plate and 5-(and-6)-carboxy-2′, 7′-dichlorodihydrofluorescein diacetate was added to final concentration of 10 μM. Cells were incubated in serum-free medium for 30 min at 37°C, protected from light, then stimulated with PM. ROS measurement was performed using the Image LIVE Green Reactive Oxygen Species Detection Kit from Molecular probes (Eugene, OR) according to the manufacturer’s instructions.

### EC permeability assays

The cellular barrier properties were analyzed by measurements of transendothelial electrical resistance (TER) across confluent human pulmonary endothelial monolayers using an electrical cell-substrate impedance sensing system (Applied Biophysics, Troy, NY) as previously described [33]. Endothelial permeability to macromolecules was monitored by Vascular Permeability Imaging Assay (Millipore, cat. #17–10398) described elsewhere [34]. Briefly, FITC-avidin tracer was added directly to the culture medium 15 min after EC stimulation with PM for 3 min before termination of the experiment. Unbound FITC-avidin was washed out with PBS (pH 7.4, 37°C), and fluorescence signal was meausred using endothelial cell monolayer imaging by immunofluorescence microscopy or Victor—X microplate reader with fluorescence reading capacity as described previously [35].

### VE-cadherin surface biotinylation assay

This assay was performed as we previously described [32]. Briefly, endothelial cell monolayers after treatment with agonists of choice for 30 min were washed with PBS at 37°C and incubated for 10 min with 5 mM Sulfo-NHS-SS-Biotin (Pierce Biotechnology, Rockford, IL) at 25°C. Subsequently, after washing of unbound Biotin, cells were lysed in 1% Triton-100 PBS, and clarified cell lysate was incubated with 60 μL of Streptavidin-agarose (Pierce Biotechnology, Rockford, IL) for 1 hr at 4°C. Beads with immobilized surface-biotinylated proteins were washed and boiled in sample buffer with 5% 2-mercaptoethanol. Samples were next subjected to western blot analysis with VE-cadherin antibody.

### Protein subcellular fractionation and immunoblotting

After agonist stimulation, cells were and lysed with cold TBS-NP40 lysis buffer (20 mM Tris pH 7.4, 150 mM NaCl, 1% NP40) supplemented with protease and phosphatase inhibitor cocktails (Roche, Indianapolis, IN). Cytosolic (soluble) and membrane/cytoskeletal (particulate) fractions were isolated as described previously [36]. Protein extracts were separated by SDS-PAGE, transferred to polyvinylidene fluoride (PVDF) membrane, and probed with specific antibodies. Equal protein loading was verified by western blot analysis of initial total cell lysates with antibody to VE-cadherin.

### Immunofluorescent staining and image analysis

Following agonist stimulation, endothelial cells were fixed in 3.7% formaldehyde solution in PBS for 10 min at 4°C, washed with PBS, permeabilized with 0.1% Triton X-100 in PBS for 30 min at room temperature and blocked with 2% BSA in PBS for 30 min. Incubation with VE-cadherin antibody was performed in blocking solution (2% BSA in PBS) for 1 hr at room temperature followed by staining with Alexa 488-conjugated secondary antibody. Actin filaments were stained with Texas Red-conjugated phalloidin diluted in the blocking solution. After immunostaining, the slides were analyzed using an inverted microscope Nikon Eclipse TE300 connected to SPOT RT monochrome digital camera and image processor (Diagnostic Instruments, Sterling Heights, MI). The images were processed with Adobe Photoshop 7.0 (Adobe Systems, San Jose, CA). For each experimental condition at least 10 microscopic fields in each independent experiment were analyzed.

#### DNA transfection

Human lung EC were transfected with PAFAH2 DNA plasmid harboring Myc-FLAG-tags (OriGene, Rockville, MD) using Lipofectamine 2000 reagent (Invitrogen, Grand Island, NY) as recommended by the manufacturer. Then, cells were treated with vehicle or PM after 24 hr of transfections and assayed for permeability measurements or other biochemical analysis.

#### Animal studies

All animal care and treatment procedures were approved by the University of Maryland and University of Chicago Institutional Animal Care and Use Committees. 8-10-week old male and female C57Bl/6j mice were purchased from Jackson Laboratories (Bar Harbor, ME). Animals were handled according to the National Institutes of Health Guide for the Care and Use of Laboratory Animals. PM was administered as suspension in saline (100 μg/mouse). Animals were anesthetized with a 0.03 ml intraperitoneal injection of ketamine (12 mg/kg). Proper anesthesia was assessed by paw and tail pinching, and additional anesthetic was administered as necessary. Animals were sacrificed at 3 hrs or 24 hrs after PM administration by exsanguination under ketamine anesthesia. After perfusion with Hank’s balanced salt buffer supplemented with 0.5 mM phenyl-methyl-sulfonyl-fluoride (PMSF) and 0.1 mM Pefa-block, lungs were snap frozen in liquid nitrogen and used for MS analysis of Tr-OxPL content.

#### Mass Spectrometry analysis of Tr-OxPLs in cell samples and lungs

The levels of Tr-OxPLs in endothelial cells and mouse lungs was determined by mass spectrometric analysis as recently described by our group [37]. Briefly, lipids were extracted by employing modified Bligh and Dyer method [38] using 2% formic acid for phase separation. Then, Tr-OxPLs content was determined by liquid chromatography electrospray ionization tandem mass spectrometry using Sciex 6500QTRAP mass spectrometer coupled with Shimadzu Nexera X2 UHPLC system.

### Statistical analysis

Results are expressed as means ± SD of three to five independent experiments. Stimulated samples were compared with controls by unpaired Student’s t-test. For multiple-group comparisons, one-way analysis of variance (ANOVA) followed by the post hoc Fisher’s test were used. P<0.05 was considered statistically significant.

## Results

### PM disrupts EC barrier via disassembly of adherens junctions

**Fig 1:**
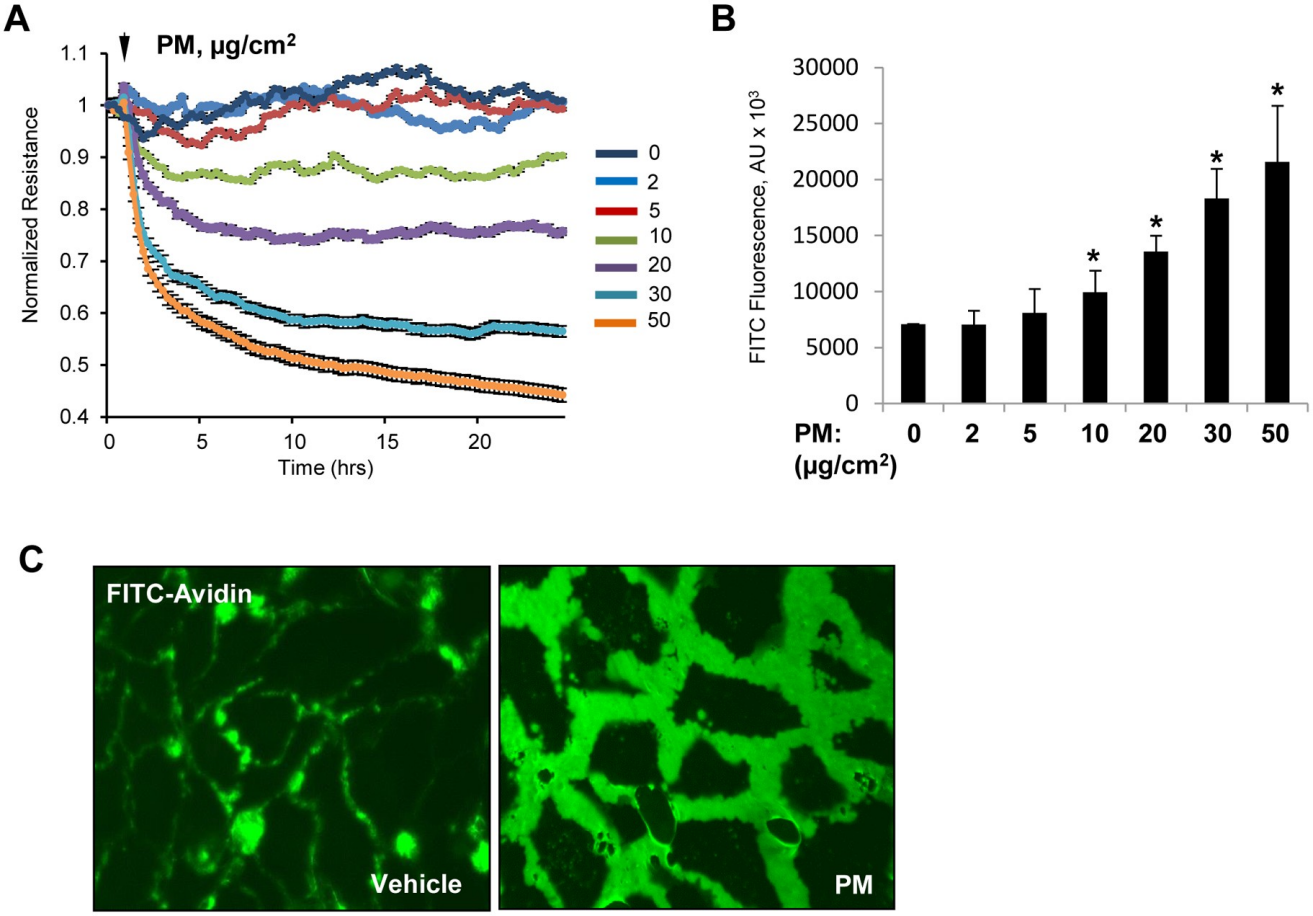
PM causes endothelial barrier disruption. (**A**) Human pulmonary lung EC were exposed to indicated concentrations of PM and TER was measured across the cell monolayers over time. (**B**) Cells grown on immobilized biotinylated gelatin were exposed to PM for 4 hr and FITC-avidin (25 μg/mL) was added for 3 min. After washing unbound FITC-avidin with PBS, FITC fluorescence was determined in Victor X5 plate reader. Normalized readings are expressed as mean ± S.D.; n = 6, *p<0.05. (**C**) Visualization of FITC fluorescence in control or PM-treated cells (20 μg/cm^2^, 4 h).

**Fig 2:**
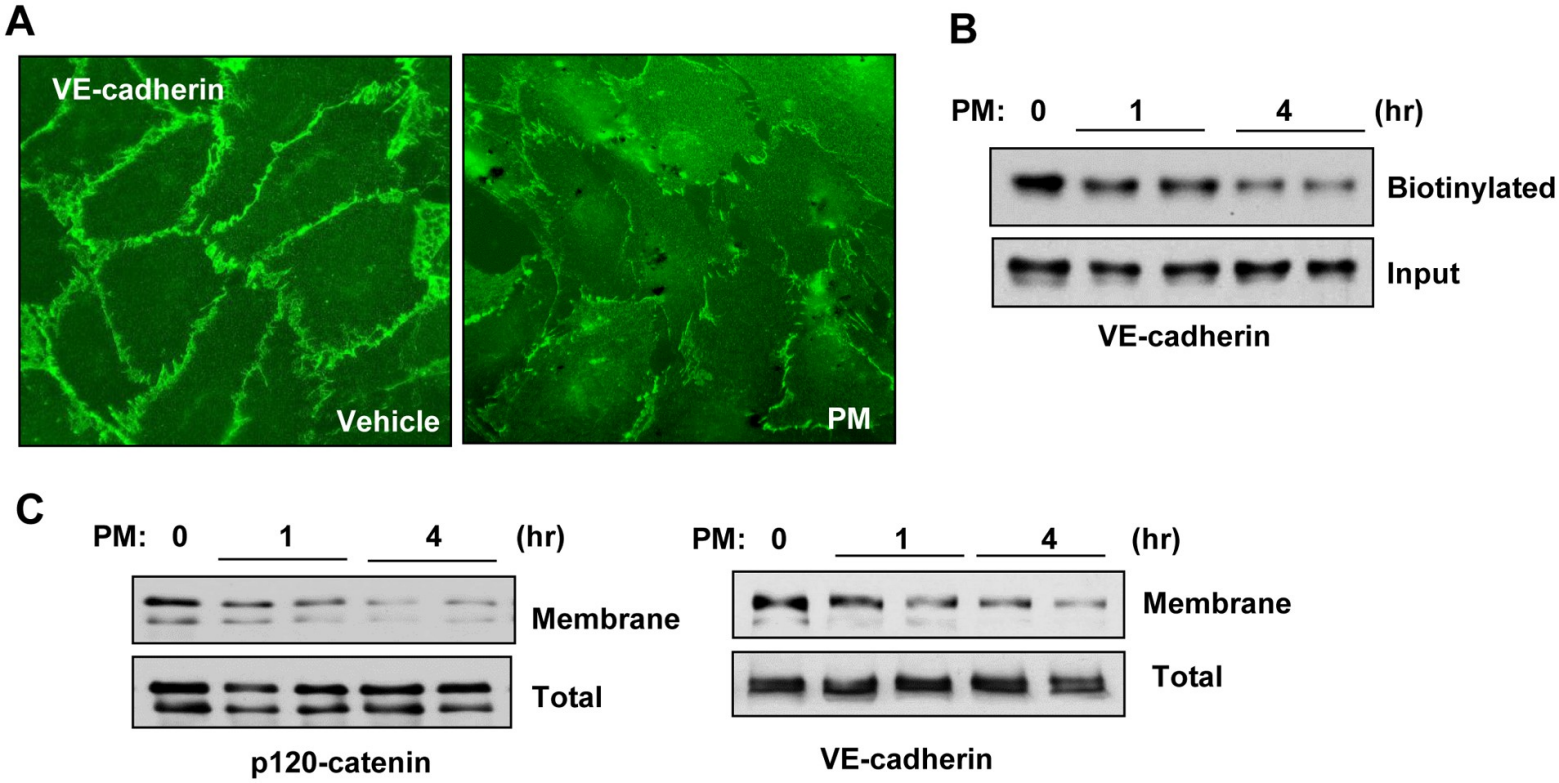
PM induces AJ breakdown. (**A**) Cells were treated with PM (20 μg/cm^2^, 1h) and cell junctions were visualized by immunostaining with VE-cadherin antibody. (**B**) Surface protein biotinylation assay was performed as described in Methods and lysates were run on Western blot to detect biotinylated and total VE-cadherin. (**C**) Membrane fractions isolated by sub-cellular protein fractionations were subjected for Western blotting against p120-catenin and VE-cadherin antibodies. The total cell lysates were used as normalization control.

We assessed the effects of PM on EC barrier function by measuring endothelial permeability with two complementary approaches: measurement of transendothelial electrical resistance (TER) using ECIS array and evaluation of endothelial cell monolayer permeability for FITC-avidin as described in Methods. Stimulation of pulmonary endothelial cell monolayers with PM caused a rapid and sustained dose-dependent decrease in TER in the dose range of 10–50 μg/ml of PM reflecting endothelial cell barrier dysfunction (Fig 1A). Accordingly, evaluation of endothelial cell monolayer permeability to macromolecules by express permeability testing (XPerT) assay developed in our group [34], showed increase in FITC fluorescence in PM-treated endothelial monolayers in a dose-dependent fashion (Fig 1B). Alternatively, immobilization of FITC-avidin on the bottoms of the wells with endothelial cell monolayers challenged with PM was visualized by fluorescence microscopy (Fig 1C). AJ play a central role in maintaining endothelial barrier integrity, and VE-cadherin is the major transmembrane protein that forms heterotypic adhesions with VE-cadherins from neighboring cells leading to establishment of endothelial monolayer barrier [39]. To test whether PM-induced EC barrier disruption was accompanied by changes in AJ integrity, we performed immunofluorescence staining of VE-cadherin in pulmonary endothelial monolayers. PM treatment caused the disappearance of VE-cadherin from cell junctions accompanied by the formation of intercellular gaps; the morphological changes reflecting disruption of cell-cell contacts and endothelial barrier failure caused by PM (Fig 2A). Analysis of PM effects on integrity of endothelial adherens junction complexes was further performed using biochemical approaches. *In situ* biotinylation assay of cell surface proteins showed a marked decrease of biotinylated VE-cadherin in PM stimulated cells suggesting VE-cadherin internalization (Fig 2B). PM also caused redistribution of adherens junction proteins VE-cadherin and p120-catenin from cell membrane fractions to cytosolic fractions, as evaluated by subcellular fractionation assay (Fig 2C). The decreased pool of biotinylated VE-cadherin and reduced membrane targeting of adherens junction proteins reflects PM-induced VE-cadherin internalization and disassembly of adherens junction complexes. These findings are consistent with PM effects on endothelial morphological changes and monolayer barrier function.

### PM-induced ROS production drives pulmonary EC barrier dysfunction

**Fig 3:**
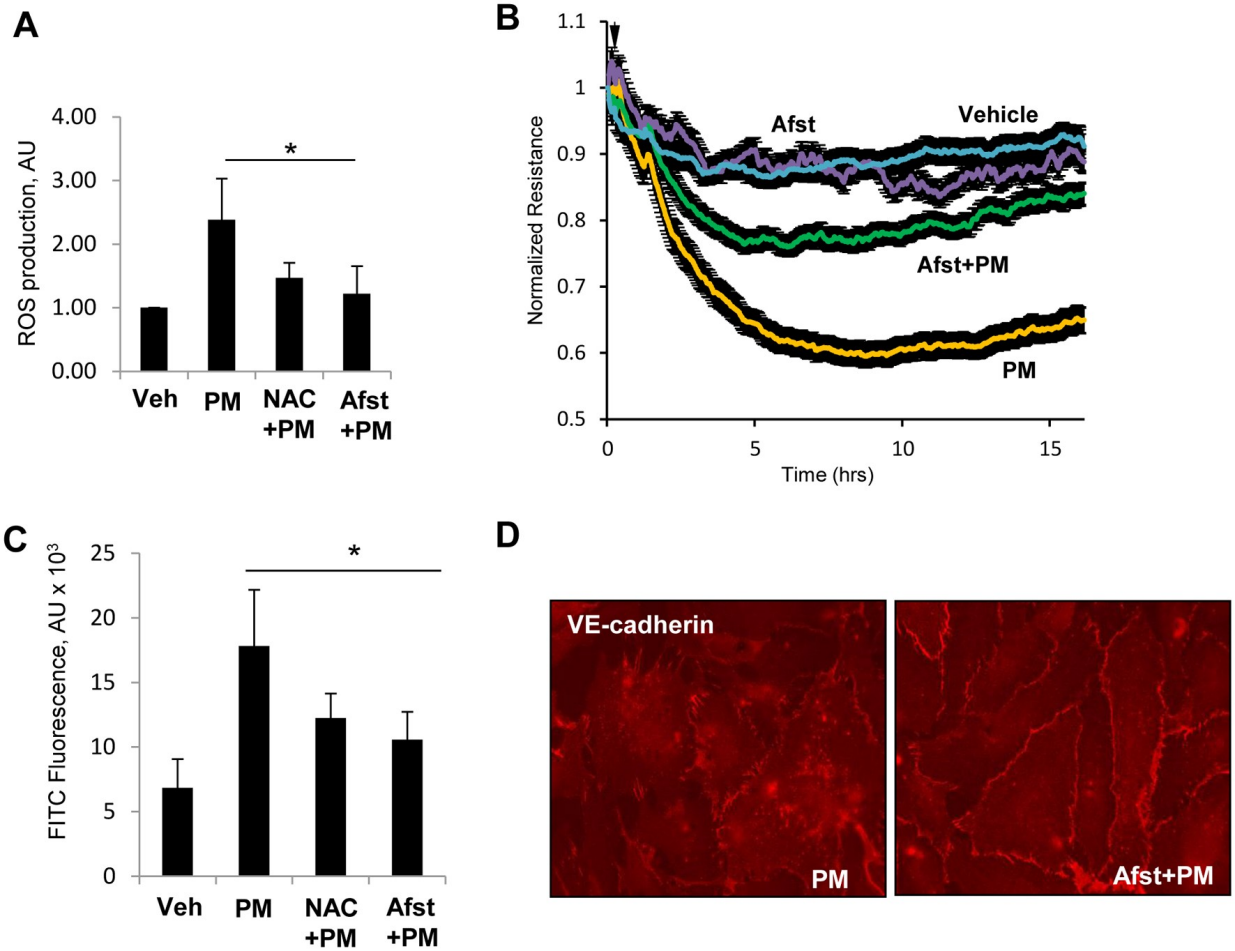
PM induces EC barrier disruption via ROS generation. (**A**) Cells were treated with PM (20 μg/cm^2^, 30 min.) with or without pre-treatment with N-acetyl cysteine (NAC, 1 mM, 30 min) or amifostine (Afst, 4 mM, 30 min) and ROS levels were measured. Normalized data are expressed as mean ± S.D.; n = 6, *p<0.05. (**B**) TER was monitored across PM-treated EC monolayers in the presence or absence of Afst. (**C**) FITC fluorescence was determined by XPerT assay described in Methods. n = 6, *p<0.05. (**D**) VE-cadherin staining of cells treated with PM with or without pretreatment with Afst.

Since earlier studies have suggested the generation of ROS as a key mechanism of PM-induced pathologies [15, 29], we sought to determine whether PM-induced endothelial dysfunction was also mediated by oxidant stress. The measurement of ROS levels by DCFDA assay showed a rapid increase in ROS following PM stimulation which was blocked by pre-treatment with known antioxidants N-acetyl cysteine (NAC) and amifostine (Fig 3A). The role of ROS production in mediating PM-induced EC barrier disruption was assessed by the measurements of endothelial permeability. The results showed that inhibition of ROS production with NAC attenuated PM-induced decrease in TER (Fig 3B). Likewise, pretreatment of EC with NAC or amifostine strongly attenuated PM-induced increase in endothelial permeability to macromolecules measured by XPerT assay (Fig 3C). The protective effects of ROS inhibition were further assessed by immunofluorescence staining and analysis of VE-cadherin positive adherens junctions. The results showed that VE-cadherin disappearance from cell junctions and formation of intercellular gaps caused by exposure to PM was abolished by pretreatment of endothelial cells with amifostine (Fig 3D).

### PM stimulates production of Tr-OxPLs by pulmonary EC

**Fig 4:**
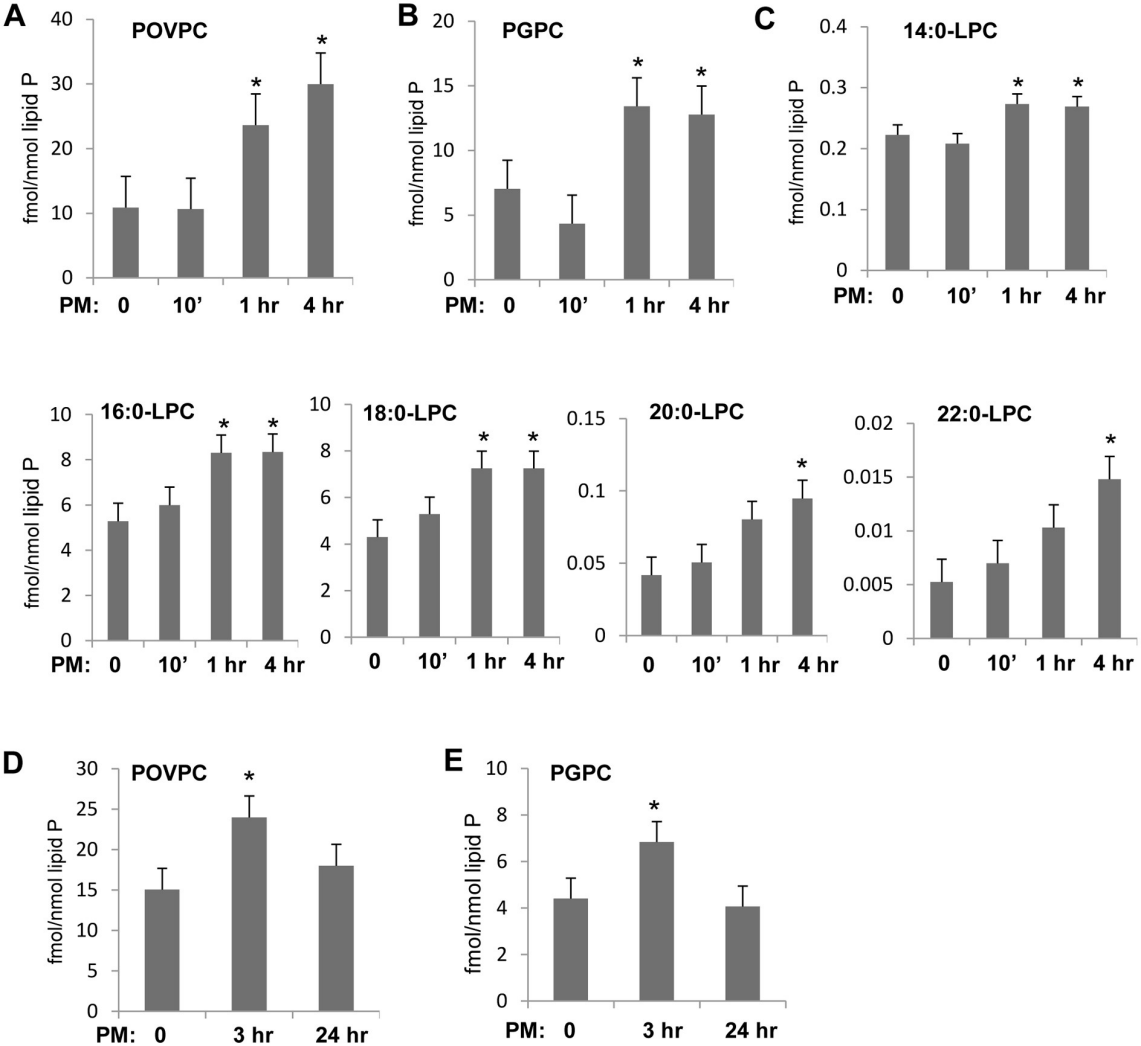
PM treatment produces Tr-OxPLs in EC and mouse lungs. (**A, B, C**) Cells were treated with 20 μg/cm^2^ of PM for indicated time points and mass spectrometry was performed as described in Methods to determine levels of POVPC (**A**), PGPC (**B**) and different variants of lyso-PC (**C**). (**D, E**) Mice were challenged with PM (100 μg/animal), and lung tissues were subjected for mass spectrometry analysis to measure POVPC (**D**) and PGPC (**E**).

To evaluate a role of Tr-OxPLs in rapid PM-induced endothelial permeability response, we used mass spectrometry approach as described in Methods. Previous studies by our group demonstrated barrier-disruptive effects of three synthetic Tr-OxPLs: POVPC, lyso-PC and PGPC, on pulmonary endothelial cells [32]. In this study, we analyzed endogenous generation of these major Tr-OxPLs in EC exposed to PM. The results showed a significant increase of POVPC, PGPC and lyso-PC variants in pulmonary EC which was observed at 1 hr and 4 hrs after PM stimulation **(**Fig 4A–4C). Further analysis of the Tr-OxPLs changes was performed in the phospholipid extracts from lung tissues of mice injected with PM. There was a significant increase in PGPC and POVPC levels in mice challenged with PM (Fig 4D and 4E). Both elevated Tr-OxPLs returned to basal levels by 24 hrs post-PM challenge.

### PM-upregulated TR-OxPLs induce EC permeability in vitro

**Fig 5:**
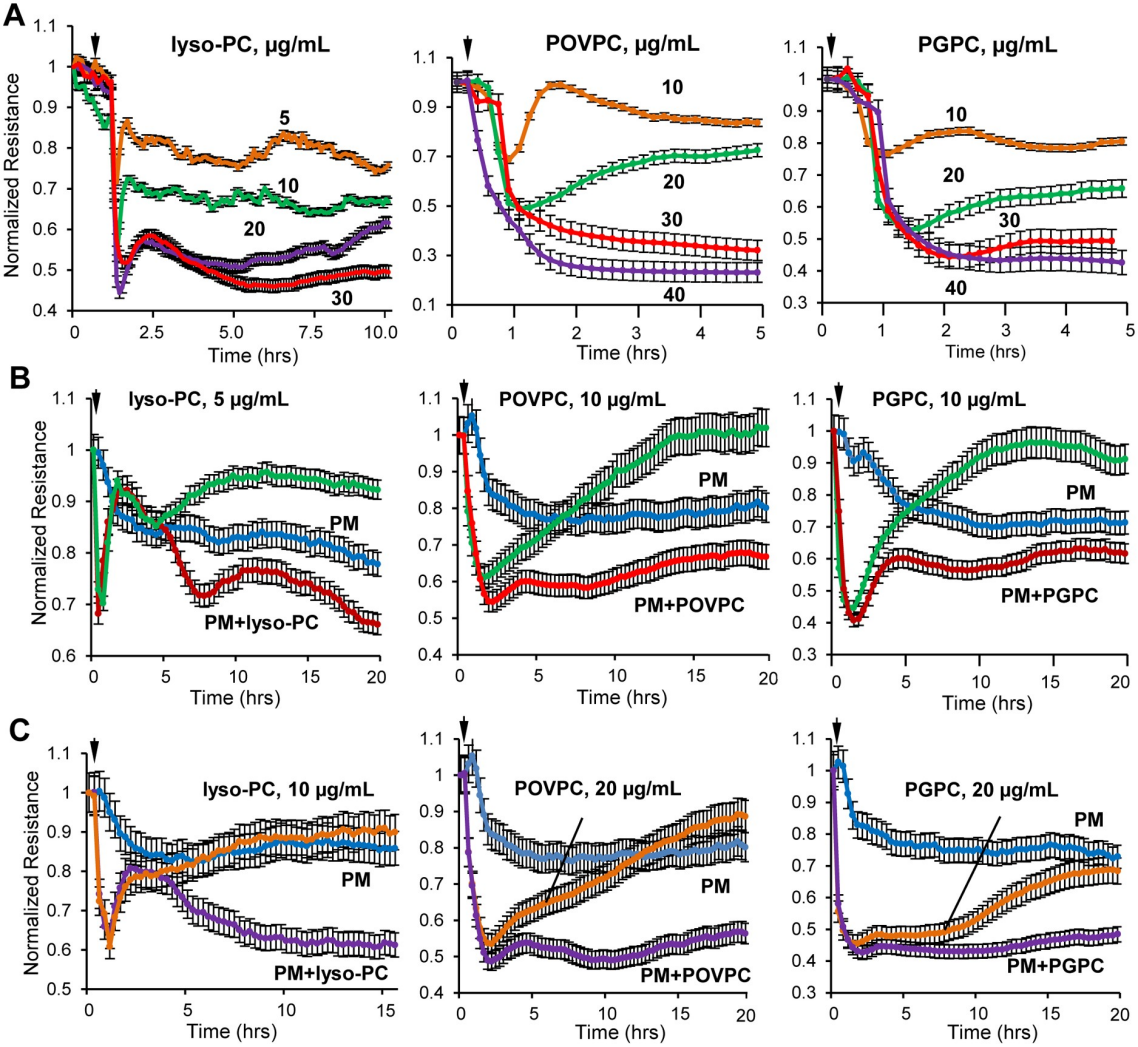
PM-produced Tr-OxPLs increase endothelial permeability. (**A**) Purified Tr-OxPLs were added to EC monolayers at indicated concentrations and TER was determined over time for lyso-PC (left panel), POVPC (middle panel) and PGPC (right panel). Next, cells were treated with sub-maximal lower (**B**) and higher (**C**) doses of Tr-OxPLs in combination with PM and endothelial barrier function was assessed by measuring TER over time. Data are expressed as mean ± S.D.; n = 5.

We studied effects of single purified truncated oxidized phospholipid species identified by MS analysis in PM-challenged endothelial cell and lung tissues. All three products: PGPC, lyso-PC and POVPC, caused monophasic, rapid and dose dependent TER decline reflecting increased EC permeability, which was observed in the 5–40 μg/ml concentration range (Fig 5A). Combination of each of Tr-OxPLs tested above and PM-0.5 at concentration which cause submaximal drop in TER, led to augmented EC barrier disruptive response (Fig 5B). These results suggest that further elevation of Tr-OxPLs generated in endothelial cells treated low PM-0.5 doses by additional supplementation of exogenous POVPC recapitulates EC permeability response to high PM-0.5 dose and, thus support the role of Tr-OxPLs as an initiating mechanism of PM-induced EC hyperpermeability.

### PM-produced Tr-OxPLs disrupt cell junctions assembly

**Fig 6:**
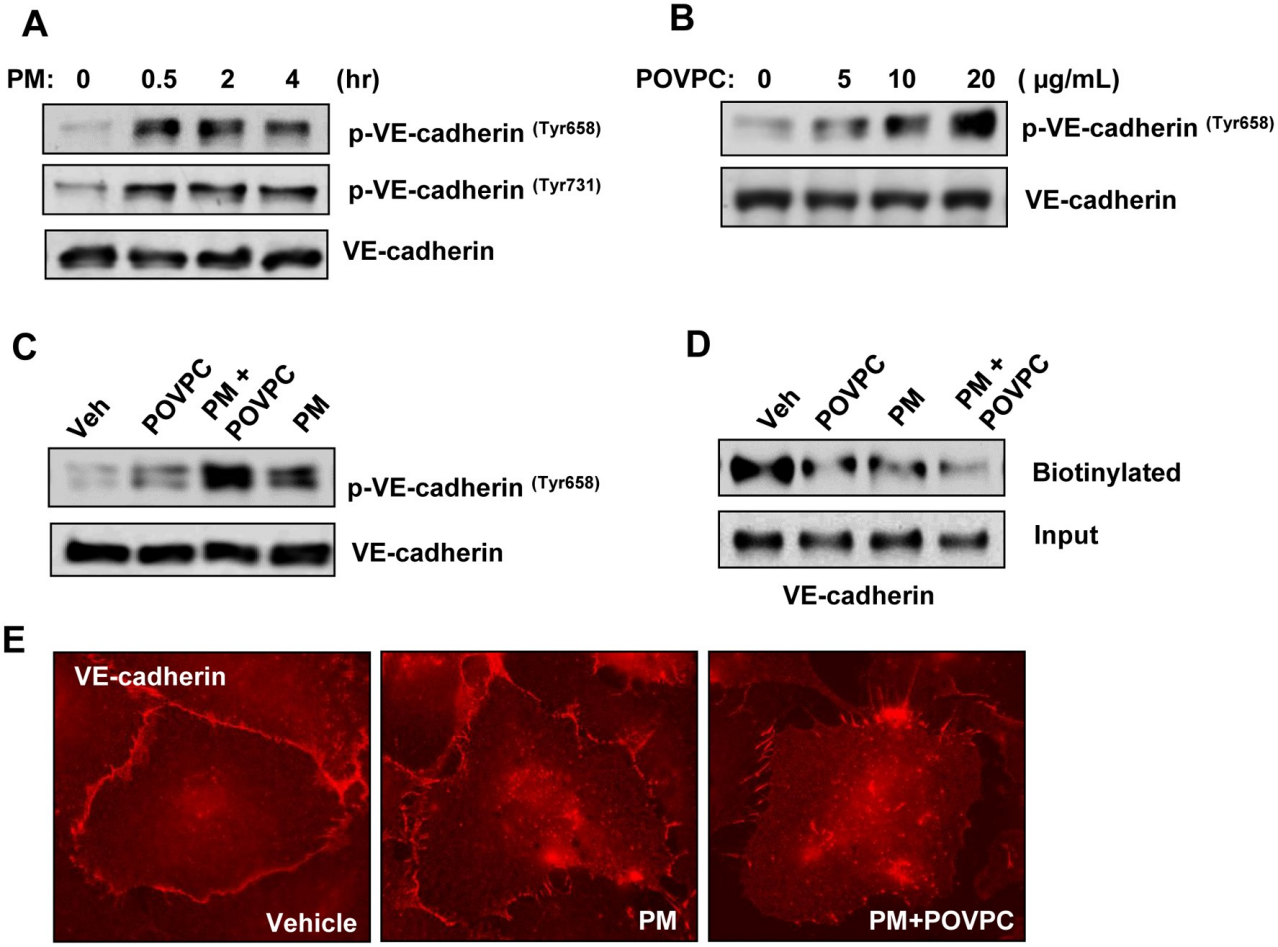
Tr-OxPLs augment PM-induced AJ disruption. (**A**) Cells were treated with PM for indicated time periods, and phospho-VE-cadherin (Tyr 658 and Tyr 731) levels were determined by Western blotting. (**B**) Cells were treated with increasing concentrations of POVPC for 30 min, and cell lysates were analyzed by Western blotting to detect phospho-VE-cadherin. (**C, D**) Cells were challenged with low dose of POVPC (10 μg/mL) alone or in combination with PM, and phospho-VE-cadherin levels (**C**) or biotinylated VE-cadherin levels (**D**) were detected by Western blotting. The total cell lysates were used as normalization control. (**E**) VE-cadherin staining of pulmonary EC following the treatment with PM alone or in combination with POVPC.

The results described above showed PM-induced production of Tr-OxPLs and adherens junction disassembly. The next studies investigated in more detail the role of PM—Tr-OxPL axis as a mechanism of rapid VE-cadherin cell junction disassembly and endothelial barrier dysfunction. VE-cadherin is a key protein regulating cell-cell interactions and endothelial barrier function. Tyrosine phosphorylation at Tyr^658^ and Tyr^731^ impairs VE-cadherin activity towards formation of adherens junctions [39]. Thus, we examined tyrosine phosphorylation of VE-cadherin at Tyr^658^ and Tyr^731^ in cells treated with PM-0.5. EC treatment with 20 μg/cm^2^ PM-0.5 caused rapid and sustained VE-cadherin phosphorylation at both sites which was observed as early as 30 min after treatment (Fig 6A). Endothelial cell treatment with POVPC caused similar pattern of VE-cadherin tyrosine phosphorylation in a dose-dependent manner (Fige 6B), as in endothelial cells treated with PM suggesting PM—TR-OxPLs mechanism of VE-cadherin phosphorylation leading to endothelial hyperpermeability. In agreement with the effects of endothelial cell co-treatment with low doses of PM and Tr-OxPL on endothelial permeability, such combined treatment resulted in augmented VE-cadherin phosphorylation (Fig 6C). VE-cadherin tyrosine phosphorylation caused by PM, Tr-OxPL or their combination was directly linked to VE-cadherin internalization detected by surface biotinylation assays (Fig 6D) and disappearance from cell-cell contacts (Fig 6E).

### Inhibition of Tr-OxPLs generation rescues PM-induced EC barrier dysfunction

**Fig 7:**
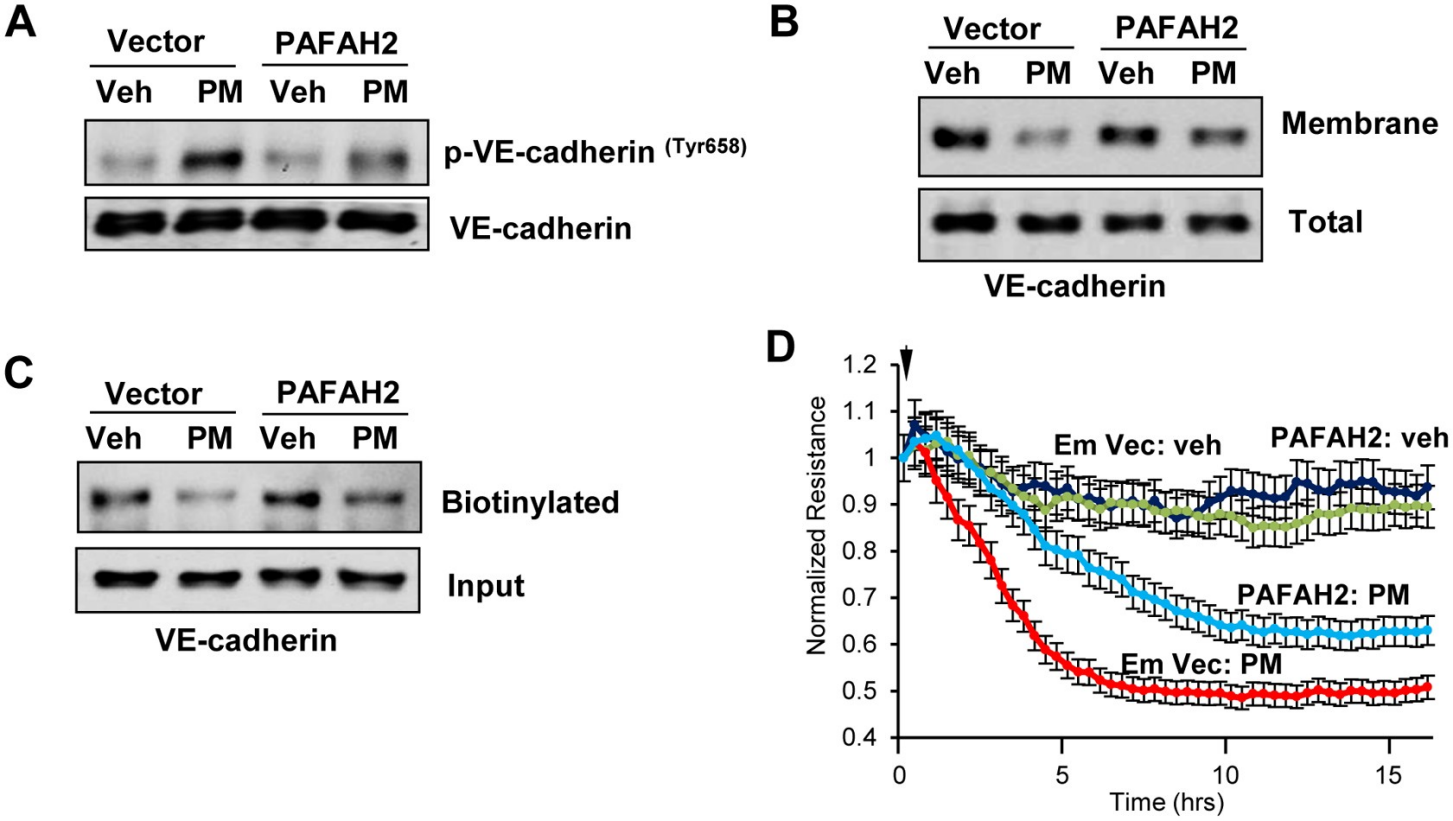
Overexpression of PAFAH2 represses PM-induced EC barrier dysfunction. (**A, B, C**) Cells were transfected with plasmid encoding human PAFAH2 or control empty vector for 24 h followed by treatment with PM (20 μg/cm^2^, 4 hr). Cell lysates were analyzed by Western blotting to determine phospho-VE-cadherin (**A**), membrane bound VE-cadherin (**B**) and biotinylated VE-cadherin (**C**) levels. Detection of VE-cadherin in total cell lysates was used as normalization control. (**D**) Cells transfected with empty control vector or PAFAH2 overexpressing plasmid were treated with PM and TER was monitored over time. Data are expressed as mean ± S.D.; n = 4.

Since our results strongly indicate that production of Tr-OxPLs is the major mechanism that mediates PM-induced endothelial cell dysfunction, we tested whether inhibition of Tr-OxPLs restores endothelial function. Hydrolysis and deactivation of truncated products of phospholipid activation via cleavage of truncated/oxidized fatty acid moiety present at *sn*-2 position of Tr-OxPL species can be accomplished by the group VII class of platelet-activating factor acetylhydrolases. Specifically, intracellular type 2 platelet-activating factor acetylhydrolase (PAFAH2) is a unique enzyme which may inactivate pro-inflammatory TR-OxPLs [40]. To evaluate a role of PAFAH2 as a potential rescue strategy to reduce the pool of PM-induced Tr-OxPLs driving endothelial barrier dysfunction, we performed ectopic expression of PAFAH2 in human pulmonary endothelial cells before their treatment with PM. PAFAH2 overexpression strongly attenuated PM-induced VE-cadherin tyrosine phosphorylation (Fig 7A). PAFAH2 overexpression also repressed PM-induced internalization and loss of VE-cadherin from plasma membrane (Fig 7B and 7C). More importantly, PAFAH2 overexpression attenuated PM-induced endothelial permeability as evidenced by recovery of TER decrease (Fig 7D).

## Discussion

PM air pollution has emerged as a severe environmental health concern with over 4 million premature deaths annually worldwide from various cardiopulmonary disorders [41]. PM is known to target the disruption of endothelial barrier but the underlying mechanisms are not known. Earlier studies have suggested the role of oxidative stress and inflammation in mediating PM-induced endothelial cell dysfunction [15, 29]. Here we report a novel paradigm of endothelial dysfunction during PM exposure as a result of PM-ROS-induced generation of Tr-OxPLs and subsequent AJ assembly disruption. Our findings demonstrate deleterious effects of PM on endothelial cells depend on elevation of ROS which triggers the production of barrier disruptive Tr-OxPLs including POVPC, PGPC and lyso-PC. These Tr-OxPLs act on VE-cadherin, a major protein of AJ complex, by inducing its phosphorylation leading to its internalization and loss from plasma membrane that ultimately disrupts endothelial cell barrier. Moreover, we also showed that inhibition of Tr-OxPLs production by PAFAH2 rescues PM-induced endothelial cell barrier disruption, suggesting a critical role of PM-ROS-TrOxPLs signaling axis during endothelial dysfunction caused by PM exposure.

In agreement with previous report of the role of ROS in PM-induced EC dysfunction [29], our data show that PM-induced ROS production is upstream of PM-caused EC hyperpermeability, since anti-oxidants NAC and amifostine largely restored endothelial barrier integrity. PM-induced generation of reactive oxygen species (ROS) is a recognized mechanism of cellular damage, inflammation and lung barrier dysfunction [42, 43]. PM induce oxidative stress via several mechanisms including activation of pro-oxidant enzymes and mitochondrial damage leading to uncontrolled ROS production [16]. In turn, oxidative stress promotes oxidation of cell phospholipids and generation of Tr-OxPLs [30]. Suppression of oxidative stress by ROS scavengers like NAC has been shown to attenuate *in vivo* oxidation of circulating lipoproteins in patients [44]. WR-1065, a bioactive thiol metabolite of amifostine, acts as free-radical scavenger and, similarly to NAC, protects cells and tissues from oxidative damage. As free-radical scavenger, amifostine demonstrated its radioprotective effects in tumor radiotherapy [45] and mitigated oxidant stress-mediated lung dysfunction in the models LPS- and ventilator- induced lung injury [46, 47]. Thus, our results further extend findings from previous studies and strongly suggest that NAC and amifostine function as anti-oxidants to mitigate PM-induced Tr-OxPL formation and abrogate deleterious consequences of elevated Tr-OxPLs in the lung.

Furthermore, we also show that PM-induced disruption of AJ complex caused by the loss of major AJ proteins VE-cadherin and p120-catenin mediates endothelial cell barrier disruption. Phosphorylation of VE-cadherin at Tyr^658^ and Tyr^731^ is known to prevent its binding to partners, p120- and β-catenin, leading to weakened AJ [48, 49]. The reduced interaction of VE-cadherin with p120-catenin also causes the internalization and loss of VE-cadherin from plasma membrane [39]. Our data show that PM or POVPC alone induced rapid tyrosine phosphorylation of VE-cadherin at Tyr-658 and Tyr-731, and VE-cadherin phosphorylation was augmented by co-treatment with PM and POVPC. Collectively, our data suggest a major role of non-contractile, adherens junction-mediated mechanism of PM-induced endothelial barrier disruption which may act in synergy with previously reported contractile mechanism of endothelial permeability mediated by RhoA and p38 MAP kinase pathways [27, 29]. Most importantly, our results revealed for the first time that PM induces the production of endothelial cell barrier disruptive Tr-OxPLs *in vitro* as well as *in vivo* that cause PM-induced endothelial dysfunction.

Lipid peroxidation products such as 4-hydroxy-2-nonenal and 8-isoprostane have been found in increased concentrations in patients with ARDS and other lung injuries and correlated with severity of disease [50–52]. However, it is important to note that not all OxPLs have deleterious effects on lung endothelium. The full length products of PAPC oxidation such as OxPAPC, PEIPC and PECPC have barrier protective and anti-inflammatory activities. Among these, a large number of studies from our group have established that OxPAPC exerts protection against endothelial barrier disruption and inflammation against a wide range of agonists in various models of *in vitro* and *in vivo* lung injuries [46, 47, 53–56]. Nevertheless, even OxPAPC at higher concentrations induces endothelial barrier disruption [57, 58]. And, Tr-OxPLs at all times negatively regulate endothelial cell function with enhanced inflammatory responses and barrier disruption [32].

Our results strongly suggest that PM-induced rapid endothelial barrier disruption occurs through the production of various Tr-OxPLs including PGPC, POVPC and a number of structural variants of lyso-PC, since each of these Tr-OxPLs species identified by mass spectrometry analysis of PM-challenged lungs and pulmonary endothelial cells caused a dose-dependent increase in endothelial permeability. Furthermore, the robust potentiating effects of POVPC in PM-induced endothelial barrier disruption strongly suggest that generation of Tr-OxPLS is essential for PM pathologic effects on lung function. These potentiating effects of POVPC were also observed with respect to PM-induced VE-cadherin phosphorylation and internalization, where minimal effects of low dose POVPC were augmented by PM co-treatment. This phenomenon may be of clinical significance. It is conceivable that as normal healthy endothelium may not become affected by low PM exposure. However, during various pathological conditions (i.e. septic inflammation) associated with elevated Tr-OxPLs, the pulmonary endothelium becomes more vulnerable to even low doses of PM. This "two-hit" model could also be valid in other aspects of lung dysfunction. For example, in healthy individuals, epithelial barrier traps PM preventing their penetration to the lung parenchyma and translocation into the circulation. But lung epithelial barrier compromise caused by infection, toxins, mechanical injury, etc., may be further exacerbated by PM leading to PM interactions with lung endothelium and propagation of ALI-associated vascular permeability and inflammation.

A definitive role of Tr-OxPLs mediated VE-cadherin phosphorylation and internalization as a mechanism of PM-induced endothelial barrier dysfunction was further supported by experiment with ectopic expression of PAFAH2, a Tr-OxPLs-specific acetyl hydrolase, which markedly attenuated PM-induced VE-cadherin phosphorylation and restored VE-cadherin presence in plasma membrane. Consistently, PAFAH2 overexpression also significantly attenuated PM-induced endothelial permeability. This finding not only confirms the vital role of Tr-OxPLs generation in exacerbation of PM-induced endothelial cell dysfunction, but also presents a promising therapeutic strategy for targeted elimination of deleterious Tr-OxPLs without global inhibition of redox-dependent processes, since ROS signaling also plays an essential role in lung physiology, innate immunity and lung recovery. In conclusion, our study demonstrates a novel pathologic mechanism of PM-induced endothelial dysfunction via production of bioactive Tr-OxPL products. These findings also highlight PAFAH2-mediated inhibition of Tr-OxPLs production as a potential therapeutic approach to alleviate PM-induced complications of lung injury and other acute cardiovascular inflammatory disorders.
